# **Measurement properties of the EQ-5D-5L and PROPr in patients with spinal muscular atrophy**

**DOI:** 10.1186/s12955-023-02204-z

**Published:** 2023-11-15

**Authors:** Richard Huan Xu, Bin Ma, Huanping Xin, Huanyu Zhang, Yan Zeng, Nan Luo, Dong Dong

**Affiliations:** 1 Department of Rehabilitation Sciences, The Hong Kong Polytechnic University, Hong Kong, China; 2 Meier Advocacy & Support Center for SMA, Beijing, China; 3 Shenzhen Research Institute, The Chinese University of Hong Kong, Shenzhen, China; 4 JC School of Public Health and Primary Care, Faculty of Medicine, The Chinese University of Hong Kong, Hong Kong, China; 5 Saw Swee Hock School of Public Health, National University of Singapore, Singapore, Singapore

**Keywords:** EQ-5D, PROMIS, Health-related quality of life, Spinal muscular atrophy

## Abstract

**Objectives:**

Spinal muscular atrophy (SMA) is a rare monogenic neuromuscular disorder caused by loss of function mutations. Measuring health-related quality of life to support economic evaluations in this population is encouraged. However, empirical evidence on the performance of preference-based measures (PBMs) in individuals with SMA is limited. This study aimed to assess the psychometric properties of the EQ-5D-5L and the Patient-Reported Outcomes Measure Information System Preference measure (PROPr) in individuals with SMA.

**Methods:**

The data used in this study were obtained via a web-based, cross-sectional survey. All participants completed the self-reporting EQ-5D-5L and PROMIS-29 questionnaires. Information about their socioeconomic and health status was also obtained. Ceiling and floor effects, convergent and divergent validity, known-group validity, and the agreement between the two measures were assessed.

**Results:**

Strong ceiling and floor effects were observed for four dimensions of the EQ-5D-5L and three subscales, including pain intensity, pain interference, and physical function, of the PROMIS-29. All hypothesized associations between EQ-5D-5L/PROMIS-29 and other neuromuscular questions were confirmed, supporting good convergent validity. Moreover, both EQ-5D-5L and PROPr scores differentiated between impaired functional groups, demonstrating good discriminative ability. Poor agreement between the EQ-5D-5L and PROPr utility scores was observed.

**Conclusions:**

The EQ-5D-5L and PROPr both appear to be valid PBMs for individuals with SMA. However, PROPr yielded considerably lower utility scores than EQ-5D-5L and their agreement was poor. Therefore, these two PBMs may not be used interchangeably in economic evaluations of SMA-related interventions.

**Supplementary Information:**

The online version contains supplementary material available at 10.1186/s12955-023-02204-z.

## Introduction

The EuroQol five-dimension questionnaire (EQ-5D) is one of the most commonly used generic preference-based measures (PBMs) to assess health-related quality of life (HRQoL), supporting health economic evaluations worldwide [1]. Currently, the EQ-5D has two versions, including the three (EQ-5D-3 L) and five (EQ-5D-5L) response-level questionnaires. The EQ-5D-5L is the updated version of the EQ-5D-3 L, which has demonstrated significant improvement in measurement properties compared to the EQ-5D-3 L [2] and is highly recommended for use in health economic evaluation [3]. Another instrument that supports health economic evaluation is the Patient-Reported Outcomes Measure Information System (PROMIS) Preference score (PROPr) measure [4]. The PROPr descriptive system is developed from the PROMIS-29 (29-item version of the PROMIS), which assesses functioning and well-being in the physical, mental, and social domains of health. The empirical evidence of assessing validity of the PROPr score in different population is very limited. For example, Hammer et al. showed that the construct validity of the PROPr is satisfactory in US general population [4].

PROPr and EQ-5D measure health utility differently. Klapproth et al. indicated that the PROPr shows a wider range of measurements at the top end of the health utility score [5]. Another study demonstrated that both EQ-5D-5L and PROPr showed high validity, but the PROPr yielded lower utility scores than the EQ-5D-5L in the general British, French, and German populations [6]. Pan et al.’s study also exhibited that the EQ-5D-5L and PROPr differed systematically on dimension and utility score levels in the US, Australian, and British populations [7]. Currently, empirical evidence on the performance of the EQ-5D and PROPr among patients with rare diseases is insufficient [8]. Regarding the EQ-5D, four out of its five dimensions focus on the physical aspects of QoL, which makes it more sensitive to measuring changes in the physical aspects of HRQoL in clinical interventions. However, the PROPr defines health in a broader spectrum, and measures not only the physical, but also the mental aspects of HRQoL, and including social well-being [9]. However, under certain conditions, the improvement of HRQoL might not be fully reflected in the restoration of physical health, and the impact of interventions on the improvement of QoL regarding mental health and social well-being is important as well [10, 11]. Nevertheless, currently, evidence on the comparison of validity between the EQ-5D and PROPr across different populations and patient groups is rare.

Spinal muscular atrophy (SMA) is a rare monogenic neuromuscular disorder caused by loss of function mutations. It results from the homozygous disruption of the survival motor neuron 1 gene by deletion, conversion, or mutation and is the leading genetic cause of infant death [12]. It is a serious condition that worsens over time. The life expectancy of individuals with SMA varies and depends on the type and severity of symptoms [13]. Recently, an increasing number of studies have used PBMs to measure HRQoL in individuals with SMA [14]. The outcomes can help medical professionals understand the clinical implications of an intervention or care plan and support economic evaluations of new health technologies or drugs to inform resource allocation for policymakers.

Economic evaluations in rare diseases can help allocate limited resources efficiently. Considering the limited number of patients, it becomes even more essential to ensure that the available resources are used in the most cost-effective way possible [15]. Additionally, economic evaluations in rare diseases play a significant role in ensuring equitable access to healthcare services. Rare diseases often face challenges in terms of access to specialized care and expensive treatments. By evaluating the economic implications of different interventions, decision-makers can make informed choices that promote fair and equitable access to treatments for all patients, regardless of their financial circumstances [16]. Further, economic evaluations provide crucial information on the economic burden of rare diseases. Rare diseases can place a substantial financial burden on individuals, families, and society as a whole. By quantifying the economic impact of these diseases, decision-makers can better understand the costs involved and develop strategies to mitigate the financial burden [17].

However, using an instrument in a population or context where it has not been used before requires testing of its psychometric properties. Currently, no previous study has assessed the psychometric properties of any PBMs in individuals with SMA. Although the EQ-5D showed satisfactory validity to support economic evaluation in different populations and patient groups [18–25], extant literature does not provide sufficient evidence that supports the performance of the EQ-5D or PROPr among individuals with rare diseases, such as SMA [14]. Therefore, this study aimed to assess the psychometric properties of the EQ-5D-5L and PROPr estimated based on the PROMIS-29 in individuals with SMA.

## Materials and methods

### Data and participants

The data used in this study were obtained via a web-based cross-sectional survey conducted in China between May and June 2022. The research team collaborated with a patient association to recruit individuals with SMA (*Meier Advocacy & Support Centre for SMA*). The inclusion criteria were as follows: (1) aged 16 years or older at the time of the study, (2) having no cognitive problems, and (3) being able to provide informed consent. Information regarding the study was sent to all eligible participants via the patient organization’s internal social network. Thereafter, all interested members were invited to join an online chat group, and a link to the study introduction and questionnaire was shared with the group. Participants could participate in the formal survey by clicking on the link provided. All participants were required to complete the EQ-5D-5L and PROMIS-29 themselves. Besides, other information about their demographics, socioeconomic status, and health status was also collected.

The research team has extensive experience in conducting online surveys with patients who have rare diseases. The data collection procedure we used is similar to what we have published in papers collaborating with other rare disease patient associations in China [24, 26, 27]. In this study, the research team closely collaborated with the patient association during data collection to ensure data quality. Detailed instructions were provided for each section of the questionnaire to ensure that respondents understood the questions. Two research assistants managed the surveying group and were available for assistance from 9 am to 10 pm, responding within 30 min. The chief manager of the patient association emphasized the importance of data quality in the surveying group throughout the data collection period. Two research assistants from the research team were responsible for checking the data quality of each response independently. They reported any completed questionnaires that took an unreasonable amount of time ( < = 15 or > = 30 min) or showed abnormal response patterns (e.g., selecting answer “1” for all questions) to the principal investigator. After a double check by the principal investigator, suspected responses were reported to the chief manager of the patient association, who then contacted the respondents and required them to re-complete the questionnaire. To prevent survey fatigue, all questionnaires had a pause function, allowing respondents to complete them at different time points. Additionally, all questions were mandatory to avoid missing data.

The study protocol and informed consent were approved by the Institutional Review Board Ethics Committee of the XXX University with Ref no.: XXXX (*details are provided in the title page*). Written informed consent was obtained from all participants.

### Measures

#### EQ-5D-5L

EQ-5D-5L consists of two sections. The first is a health state classification system, which comprises five dimensions (mobility, self-care, usual activities, pain/discomfort, and anxiety/depression), with a five-response level option ranging from “no problem” to “extreme problems.” All health states described by the classification system can be summarized as utility scores ranging from 0 (death) to 1 (full health); a negative score indicates a health state worse than death. In this study, the EQ-5D-5L utility score was estimated based on Chinese preference weights. The second section is the Visual Analog Scale (EQ-VAS). It ranges from 0 (worst imaginable health) to 100 (best imaginable health), which represent a person’s global assessment of health.

#### PROMIS-29 and PROPr

The PROMIS-29, version 2.0, comprises 29 items under seven core domains, including physical function, fatigue, pain, anxiety, depression, sleep disturbance, and the ability to participate in social roles and activities. Each item of the PROMIS-29 is rated on a five-point Likert scale ranging from “never” to “very much,” except for the pain intensity domain, which is a VAS with a score ranging between 0 and 10. The domain score, expressed as either the T-score or the theta score, can be calculated using a web-based calculator (healthmeasures.net/score-and-interpret/calculate-scores). Higher scores indicate more symptoms and impairment in the depression, anxiety, pain interference, fatigue, and sleep disturbance subscales, whereas lower scores indicate weaker physical and social functioning.

PROPr in this study was calculated based on the six domain scores (depression, fatigue, pain, physical function, sleep disturbance, and ability to participate in social roles and activities) of PROMIS-29 and another domain, cognition. As cognition is not a part of the PROMIS-29, it was calculated based on the scores of the six PROMIS-29 domains. The formula used to calculate the cognition score was introduced by Dewitt et al. [28].The theta scores of all seven PROPr domains were then fed into the online PROPr utility function to obtain a PROPr utility score [29].

#### The pediatric quality of life Inventory

The Pediatric Quality of Life Inventory (PedsQL) 4.0 Generic Core Scales is an HRQoL measure that has demonstrated good reliability and construct validity in various rare disease-specific populations [30–32]. It is typically used among children and adolescents aged 2–18 years but has now been extended for use with adults [33]. The benefit of using the PedsQL in patients with rare diseases is that it can provide consistent results to capture and compare the changes in HRQoL in a life-long process, given that nearly all rare diseases are congenital.

#### SMA independence scale

The 22-item SMA independence scale (SMAIS) upper-limb module is a newly developed instrument that measures the level of assistance required to perform daily activities [34]. A higher score indicates a better ability to perform daily activities in individuals with SMA.

### Statistical analysis

A descriptive analysis was used to describe the patients’ background characteristics and health status. The EQ-5D-5L and PROMIS-29 profiles, including mean, standard deviation, median, score range, ceiling (percentage of highest possible scores), and floor effects (percentage of lowest possible scores), were presented. The ceiling (floor) effects were moderate (10–15%), minor (5–10%), and negligible (< 5%) [35].

Construct validity, including convergent and divergent validity, was assessed using hypothesis testing. We hypothesized correlations (1) between domains of EQ-5D-5L and PROMIS-29 (e.g., EQ-5D “Mobility” and PROMIS-29 “Physical function”); (2) between EQ-5D-5L dimensions/PROMIS-29 subscales and PedsQL core questionnaire (e.g., EQ-5D “Mobility” and PedsQL “Physical functioning” and PROMIS-29 “Physical function” and PedsQL “Physical functioning”); and (3) between EQ-5D-5L utility score/PROPr and SMAIS score. Spearman’s correlation coefficient (ρ) was used to assess the strength of the hypotheses (weak, ρ ≤ 0.35; moderate, 0.36 ≤ ρ ≤ 0.5; or strong, ρ > 0.5) [36]. Additionally, agreement between the EQ-5D-5L utility score and PROPr was assessed based on the intraclass correlation coefficient (ICC, > 0.7, satisfactory) and Bland-Altman (B-A) plot [37]. A bootstrap method (resamples = 1,000) was used to calculate the robust 95% confidence interval (95% C.I.) of the coefficient.

Known-group validity was examined using the Wilcoxon signed-rank test or Kruskal-Wallis test based on the individual’s reported symptoms and types of SMA [38]. We hypothesized that individuals with clinical symptoms would be more likely to report a lower utility score. We also calculated the effect size using the formula $r=z/\sqrt{N}$ (dividing the z value by the square root of N), to examine the relative efficiency of the measures in differentiating individuals whose conditions differed: r < 0.3 indicates a small effect, between 0.30 and 0.5 indicates a moderate effect, and r ≥ 0.5 indicates a large effect [39].

A multivariate linear regression model adjusted for demographics (sex and age) and health status (duration and SMA types) was used to further evaluate the discriminative ability of the EQ-5D-5L and PROMIS-29 in predicting the change in overall health status (EQ-VAS) at both the utility score and dimension levels. The R software (R Foundation, Austria) was used to perform all analyses, and the significance level was set at p ≤ 0.05.

## Results

### Sample characteristics

Table 1 presents the background characteristics of 137 individuals with SMA (response rate = 137/142 = 96.5%). Among them, 52.6% (n = 72) were female, 19% were younger than 20 years, and 83.9% were urban residents. Regarding SMA type, more than 40% were Type I SMA, 55% were Type II, and only 2.2% were Type III. The mean duration of SMA was approximately 15 years, with a range of 1–46 years. The refusal rate and proportion of missing values of EQ-5D-5L and PROMIS-29 were zero, demonstrating satisfactory accessibility.

**Table 1:** Participant’s background characteristics (n = 137)

	n	%
**Sex**		
Male	65	47.4
Female	72	52.6
**Age group**		
16 ~ 20	26	19
21 ~ 30	67	48.9
> 30	44	32.1
**Family registry**		
Urban	115	83.9
Rural	22	16.1
**Type of SMA**		
Type 1	58	42.3
Type 2	76	55.5
Type 3	3	2.2
**Determination of SMN2 copy number**		
Yes	78	56.9
No	29	21.2
Not clear	30	21.9
**Duration (year), mean (SD) [range]**	14.9 (10.7)	[1 ~ 46]

### Ceiling and floor effects

Regarding EQ-5D-5L, strong ceiling and floor effects were observed for most dimensions (Table 2), with pain/discomfort showing a floor effect of 47.4%, whereas self-care showed a ceiling effect of 49.6%. Only mobility and usual activities exhibited no ceiling and floor effects, respectively. None of the participants reported either the best or worst health status measured using the EQ-5D-5L. Regarding PROMIS-29, no subscales demonstrated ceiling effects; however, pain interference and physical function demonstrated a strong floor effect, whereas some mental health subscales exhibited mild floor effects.

**Table 2:** Profile of EQ-5D and PROMIS-29

Variable	Ceiling effect (%)	Floor effect (%)	Mean	Standard deviation	Median	Range
**EQ-5D-5L dimensions**						
Mobility	11.7	65.7	–	–	–	–
Self-care	49.6	22.6	–	–	–	–
Usual activities	29.2	8	–	–	–	–
Pain/discomfort	30.7	47.4	–	–	–	–
Anxiety/Depression	35.8	28.5	–	–	–	–
Best health (11,111)	0.0	–	–	–	–	–
Worst health (55,555)	–	0.0	–	–	–	–
**EQ-5D utility score**	0.7	0.7	0.27	0.25	0.3	-0.35–0.75
**EQ-VAS**	1.5	2.2	57.6	23.5	57.5	7 ~ 100
**PROMIS-29 subscales**						
Anxiety/Fear	0.7	16.8	57.7	10.3	57.5	40.3–81.4
Cognition	0.7	0.7	49.9	9.9	49.3	25.2–75.9
Depression/Sadness	3.6	18.2	58.2	10.6	58.9	41–79.3
Fatigue	2.2	3.6	54.4	8.9	53.2	33.7–75.8
Pain intensity VAS	0.7	27	2.4	2.3	2	0–10
Pain interference	2.2	32.1	53.5	9.6	55.7	41.6–75.6
Physical function	2.2	45.3	29.1	8.2	26.5	22.6–57
Sleep disturbance	0.7	2.9	49.3	6.5	50.1	32–63.9
Social roles and activities	2.9	15.3	41.3	9.1	40.3	27.5–64.2
**PROPr**	0.7	0.7	0.25	0.16	0.22	0.04–0.93

### Distribution of EQ-5D-5L utility score and PROPr

**Fig. 1:**
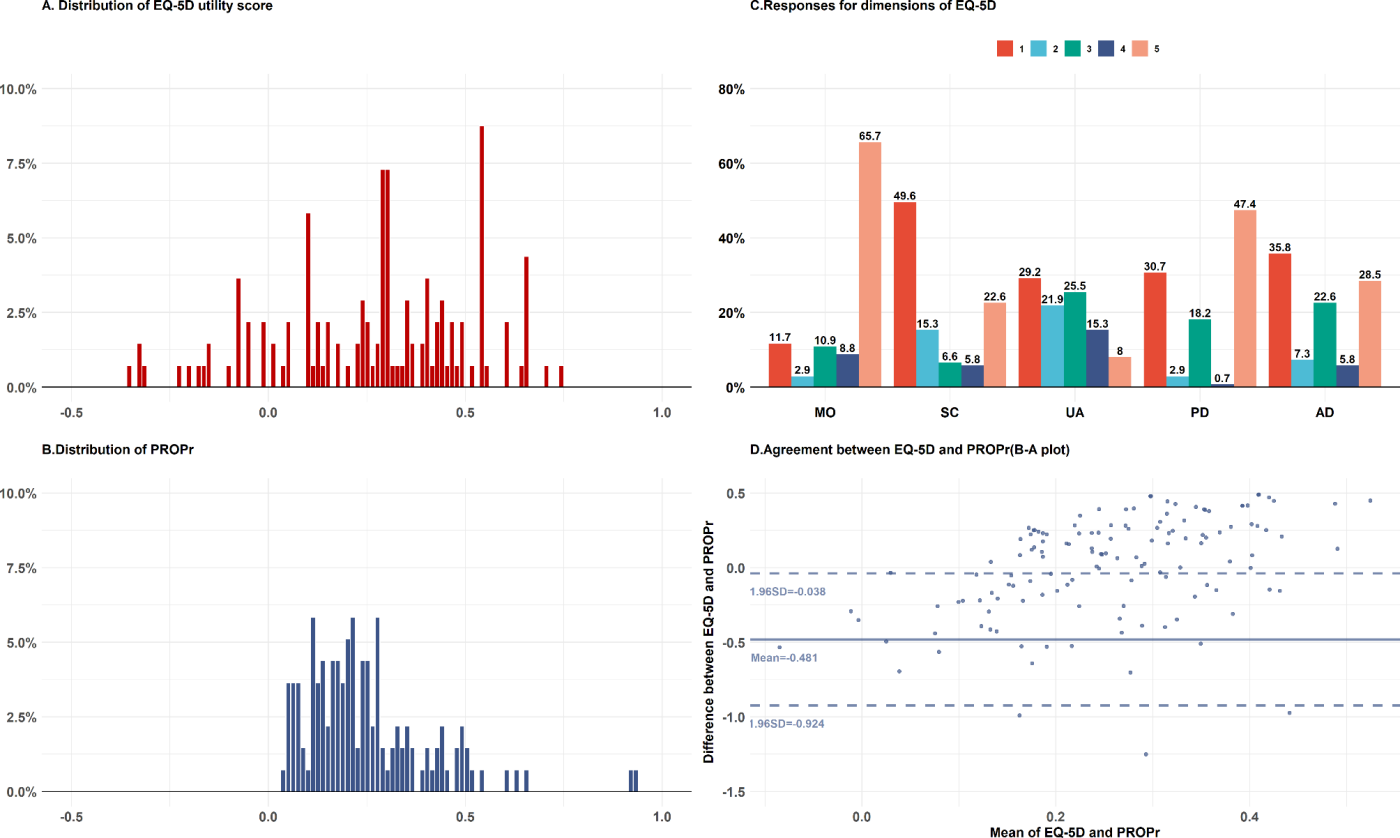
Score distribution, dimension proportion and agreement between the EQ-5D and PROPr

Table 2 shows that individuals with SMA reported a slightly lower PROPr than the EQ-5D-5L utility score (0.25 vs. 0.27). Parts A and B of Fig. 1 demonstrate that the distribution of the two scores is different, where the score range of the EQ-5D utility value was broader than the PROPr; the distribution of PROPr was skewed toward zero. Part C of Fig. 1 presents that most individuals with SMA experienced severe problems with mobility (65.7%) and pain/discomfort (47.4); however, approximately 35.8% reported having no problems regarding anxiety/ depression.

### Convergent and discriminant validity

All hypothesized associations between EQ-5D-5L and PROMIS-29 and the associations between PedsQL and EQ-5D-5L/PROMIS-29 were confirmed (Table 3). The correlation coefficient ranged between approximately 0.4 and 0.7 for the association between physical health dimensions of the EQ-5D-5L and PROMIS-29; however, regarding the mental health dimension, the correlation was significant but weaker. The convergent correlation coefficients between the EQ-5D-5L and PedsQL ranged between 0.23 and 0.83, which were weaker than those between the PROMIS-29 and PedsQL, with correlation coefficients between 0.43 and 0.82. Additionally, an ICC of 0.4 indicated that the agreement between the EQ-5D-5L utility score and PROPr was poor, and the PROPr showed a stronger correlation with SMAIS than the EQ-5D-5L utility score. The B-A plot graphically described the systematic differences between EQ-5D-5L and PROPr utility scores (Part D of Fig. 1).

**Table 3:** **Convergent validity between EQ-5D-5L and PROMIS**

Items/Dimensions	Correlation coefficient (95% C.I.)
**EQ-5D dimensions and PedsQL corequestionnaire**	
EQ-5D Mobility vs. PedsQL Physical functioning	-0.66 (-0.73, 0.55) ^***^
EQ-5D Usual Activity vs. PedsQL Social functioning	-0.31 (-0.45, -0.14) ^***^
EQ-5D Pain/discomfort vs. PedsQL item hurt or ache	-0.23 (-0.28, -0.05) ^***^
EQ-5D Anxiety/depression vs. PedsQL Emotional functioning	-0.23 (-0.41, -0.05) ^**^
EQ-5D Self-care vs. PedsQL item hard to take bath or shower	-0.83 (-0.89, -0.73) ^***^
**PROMIS-29 and PedsQL core questionnaire**	
PROMIS Physical function vs. PedsQL Physical functioning	0.65 (0.51, 0.76) ^***^
PROMIS Fatigue vs. PedsQL physical functioning	-0.41 (-0.53, -0.26) ^***^
PROMIS Anxiety/Fear vs. PedsQL Emotional functioning	-0.82 (-0.86, -0.77) ^***^
PROMIS Depression/Sadness vs. PedsQL Emotional functioning	-0.82 (-0.88, -0.75) ^***^
PROMIS Cognition vs. PedsQL Emotional functioning	0.81 (0.74, 0.86) ^***^
PROMIS Social roles and activities vs. PedsQL Social functioning	0.62 (0.46, 0.73) ^***^
PROMIS Pain intensity VAS vs. PedsQL item hurt or ache	-0.43 (-0.56, -0.26) ^***^
PROMIS Pain interference vs. PedsQL item hurt or ache	-0.49 (-0.61, -0.35) ^***^
PROMIS Sleep disturbance vs. PedsQL item Trouble sleeping	-0.71 (-0.67, -0.47) ^***^
**EQ-5D Utility score, PROPr and SMAIS**	
EQ-5D Utility score vs. SMAIS	0.28 (0.11, 0.41) ^***^
PROPr vs. SMAIS	0.38 (0.2, 0.49,) ^***^
**EQ-5D dimensions and PROMIS-29**	
EQ-5D Mobility vs. PROMIS Physical function	-0.72 (-0.78, -0.61) ^***^
EQ-5D Self-care vs. PROMIS Physical function	-0.67 (-0.77, -0.53) ^***^
EQ-5D Usual activities vs. PROMIS Social roles and activities	-0.3 (-0.46, -0.12) ^***^
EQ-5D Pain/discomfort vs. PROMIS Pain intensity VAS	0.51 (0.38, 0.62) ^***^
EQ-5D Pain/discomfort vs. PROMIS Pain interference	0.41 (0.25,0.55) ^***^
EQ-5D Anxiety/depression vs. PROMIS Anxiety/Fear	0.21 (0.02,0.38) ^**^
EQ-5D Anxiety/depression vs. PROMIS Depression/Sadness	0.18 (0.01,0.35) ^*^
**Agreement between EQ-5D utility score and PROPr (ICC)**	
EQ-5D Utility score vs. PROPr	0.4 (0.31,0.57)

* p < 0.05; ** p < 0.01; *** p < 0.001

Table 4 presents the results of the multivariate linear regression model adjusted for sex, age, and type of SMA. R-square and BIC demonstrated that the PROPr and PROMIS-29 subscale models performed better than the EQ-5D utility score and dimension models.

**Table 4:** Coefficients (95% C.I.) of linear regression analysis between EQ-VAS and EQ-5D and PROMIS adjusted by demographics

	EQ-5D utility score model	EQ-5D dimension model	PROPr model	PROMIS dimension model
**EQ-5D**				
Utility score	-13.05(-29.66,3.56)	002D	–	–
Mobility	–	-1.34(-4.9,2.23)	–	–
Self-care	–	1.98(-1.39,5.35)	–	–
Usual activities	–	2.45(-1.15,6.05)	–	–
Pain/Discomfort	–	0.55(-1.88,2.98)	–	–
Anxiety/depression	–	0.98(-1.55,3.51)	–	–
**PROMIS**	–	–	–	–
PROPr	–	–	57.04(33, 81.1)	–
Anxiety/Fear	–	–	–	-2.42(-49.91,45.07)
Cognition	–	–	–	-0.32(-8.18,7.55)
Depression/Sadness	–	–	–	1.79(-23.28,26.85)
Fatigue	–	–	–	-0.87(-1.59,-0.15)
Pain intensity VAS	–	–	–	-1.63(-37.38,34.12)
Pain interference	–	–	–	0.52(-10.37,11.41)
Physical function	–	–	–	-0.14(-0.73,0.46)
Sleep disturbance	–	–	–	-3.4(-82.54,75.74)
Social roles and activities	–	–	–	0.8(-12.04,13.64)
**Gender - Male**	-2.35(-10.54,5.83)	-2.93(-11.13,5.26)	-3.73(-11.39,3.93)	-5.89(-13.62,1.84)
**Age**	-0.11(-0.71,0.49)	-0.11(-0.72,0.51)	-0.11(-0.67,0.45)	-0.08(-0.62,0.47)
**Duration**	-0.05(-0.46,0.35)	-0.09(-0.5,0.33)	0.04(-0.34,0.42)	0.08(-0.31,0.47)
**Type T2**	-6.72(-15.75,2.31)	-12.79(-23.3,-2.27)	-8.81(-17.15,-0.47)	-9.21(-18.44,0.01)
**Type T3**	20.66(-7.6,48.93)	8.77(-21.39,38.93)	16.34(-9.97,42.64)	14.42(-11.81,40.65)
**Adjuste R** ^**2**^	0.03	0.03	0.15	0.2
**BIC**	1273.56	1287.74	1254.66	1277.28

### Known-group validity

**Fig. 2:**
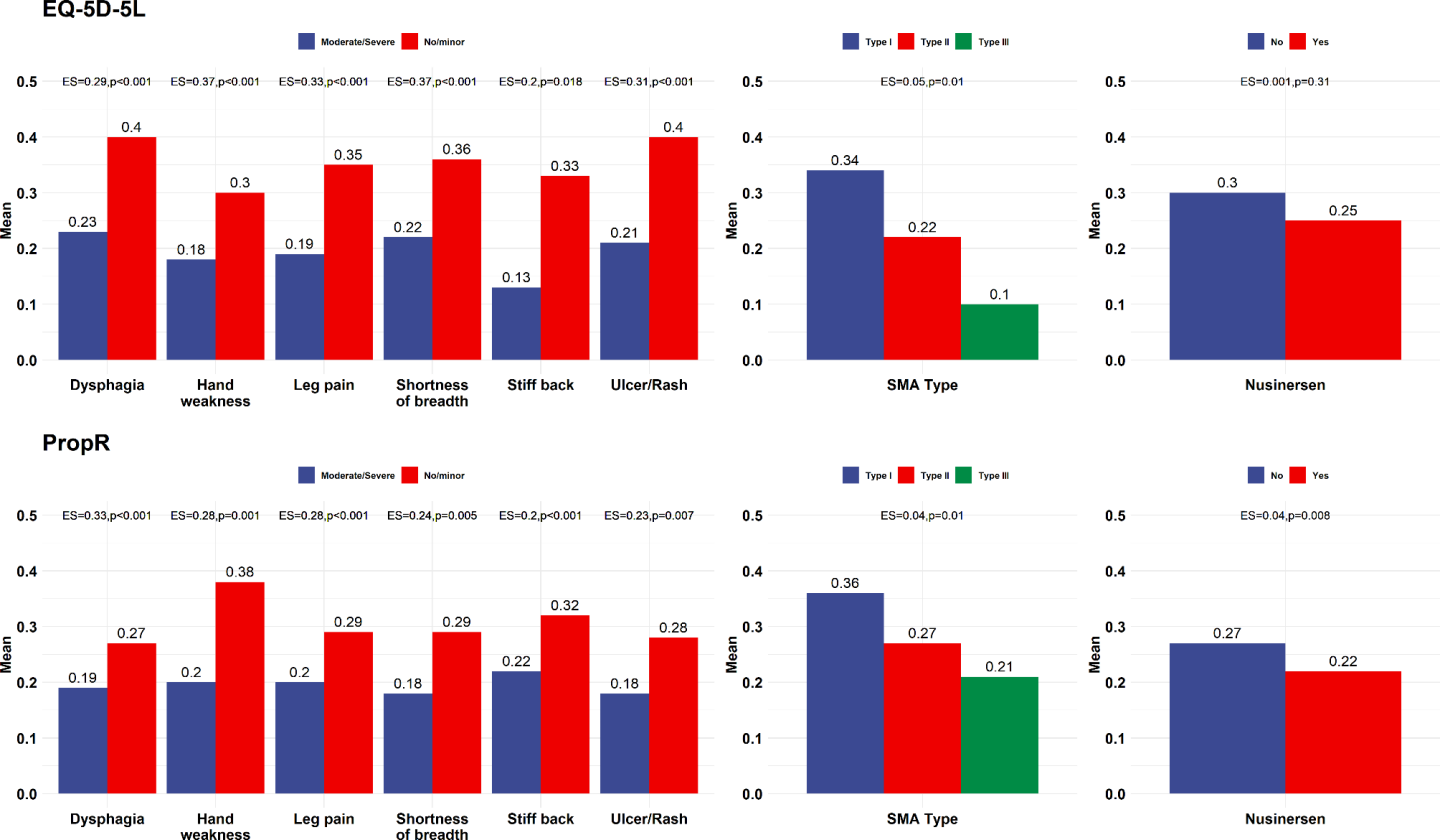
Known-group validity of the EQ-5D-5L utility score and PROPr

Figure 2 demonstrates the known-group construct validity of PROPr and EQ-5D-5L for the symptom groups. The outcomes of the Wilcoxon rank test showed that both EQ-5D-5L and PROPr could significantly differentiate between individuals with no/minor and moderate/severe symptoms. The effect size indicated that EQ-5D-5L had a larger efect size than PROPr regarding most symptoms and SMA types. Full results are presented in the appendix (Table A1).

## Discussion

To the best of our knowledge, this is the first study to compare the psychometric properties of the PROPr measured using PROMIS-29 and EQ-5D-5L in individuals with SMA. We found that both EQ-5D-5L and PROPr are valid utility measures for assessing the HRQoL in individuals with SMA. This finding contributed to existing knowledge regarding the performance of existing generic preference-based instruments in measuring HRQoL to support the economic evaluation of rare neuromuscular diseases. The findings of this study contribute to a broader understanding of the measurement properties of two major PBMs, particularly the PROPr, and their performance using various psychometric methods. This knowledge aids in the continuous efforts to enhance the measurement of HRQoL in SMA and other rare neuromuscular diseases; and leads to better-informed decisions and improved healthcare outcomes for patients. Furthermore, the findings of this study hold significant implications from a policy-making perspective. Accurately measuring HRQoL using appropriate instruments is crucial for conducting economic evaluations of healthcare interventions for SMA. By demonstrating the performance of PROMIS-29 and EQ-SD-5L in measuring HRQoL in SMA, this study provides decision makers with valuable information for selecting the most suitable instruments and outcome measures. This ensures that economic evaluations effectively capture the impact of interventions on the HRQoL of individuals with SMA.

In this study, the mean PROPr score was slightly lower than the EQ-5D-5L utility score, which is consistent with previous studies on the general population [4, 6] and patients [40]. Both PROPr and EQ-5D-5L utility scores showed no ceiling or floor effects, which is at odds with a previous study that assessed the psychometric properties in the general population of three Western countries [6]. Absence of ceiling effect means that the instruments would be sensitive to improvements in healthier patients. Nevertheless, Fig. 1 suggested that, compared to the EQ-5D, a higher proportion of SMA patients reported a close-to-worst health status using PROPr measure, which needs to be examined in future longitudinal studies.

At the dimension/subscale level, despite no ceiling effects observed for the PROMIS-29 subscales, all dimensions of the EQ-5D-5L, except for mobility, showed a strong ceiling effect. This is not surprising as ceiling effects were observed for EQ-5D-5L in many previous studies. Nevertheless, in this study, we found that four out of five dimensions of the EQ-5D-5L showed a strong floor effect, which has rarely been reported. For instance, a recent systematic review indicated that 43 out of 48 studies reported no floor effect problems in the EQ-5D-5L [41]. Our previous studies on the use of EQ-5D-5L in patients with rare disease (e.g., spinal and bulbar muscular atrophy or hemophilia) also exhibited an absence of floor effects on EQ-5D-5L dimensions [23, 24]. Additionally, although some studies, such as those by Golicki et al. [42] and Kohler et al. [43], reported a floor effect on mobility, self-care, and usual activities of the EQ-5D in patients with stroke or after cesarean section, no study has reported a strong floor effect on the mental health dimension of the EQ-5D-5L. it might because that around half of participants with type I SMA, which is the most common but severe form in our survey and the mean duration is around 15 years. Long-term symptoms, such as muscle weakness and trouble breathing lead to a poorer mental health.

The poor agreement between the PROPr and EQ-5D-5L utility scores is expected. A previous study indicated that the narrower range of values in PROPr is a product of its higher minimum value and the best health status, which has a utility score of < 1 [7]. Further, the correlation regarding the mental-health dimensions between EQ-5D-5L and PROMIS-29 was weak. However, compared to EQ-5D-5L, a stronger association of mental health dimensions of PedsQL with PROMIS-29 was identified, which indicated that PROMIS-29 might be more sensitive to capturing changes in the mental health aspects of HRQoL than EQ-5D-5L in individuals with SMA. This may also explain the poor agreement between the EQ-5D-5L and PROPr utility scores among individuals with SMA compared to previous studies that focused on general population [5, 6]. A plausible explanation might be that PROPr was estimated based on American people’s preferences, resulting in a systematic bias in the assessment; a higher proportion of individuals rated their health status at the lower-end of the PROPr utility scores. Additionally, PROPr was designed for the wider PROMIS system, rather than just for PROMIS-29, which resulted in the truncated PROPr utility scores [7].

The results of the multivariate linear regression models confirmed that the PROMIS-29 showed a better ability to predict the overall health status than the EQ-5D-5L in terms of both dimension and utility score levels. This is reasonable because PROMIS-29 includes more health-related domains than the EQ-5D-5L and is able to capture the impact of social well-being (e.g., social roles and activities) on the HRQoL that EQ-5D may not detect [25]. This study showed that the difference between the EQ-5D and PROPr might be bigger among individuals with rare diseases than among those with more common disease; however, further investigations to confirm this finding in the other rare disease populations are needed.

The known-group validity of EQ-5D-5L and PROPr is supported by our symptom impact analysis. Individuals reported moderate/severe symptoms for both utility score and PROPr, which were significantly different from no/minor for all health conditions, similar to previous studies [4, 40]. However, the EQ-5D-5L utility score yielded a larger effect size for most health conditions than the PROPr. This is pertinent because, compared to the EQ-5D-5L, PROMIS-29 assesses a broader scope of domains related to mental health and social well-being, and many of these (e.g., fatigue, sleep disturbance, and depression) are highly relevant to individuals with SMA.

Assessing the content validity of PBMs is extremely important. Previous studies consistently highlight the low content validity of generic PBMs, such as EQ-5D, across various patient populations [20, 44–46]. Therefore, it is suggested that using condition-specific PBMs may be more appropriate. However, currently there is no SMA-specific PBM available, which presents a significant challenge in accurately capturing the unique impact of this disease on patients’ lives. A new condition-specific PBM called DMD-QoL [47] has been developed for individuals with Duchenne Muscular Dystrophy, a condition similar to SMA. DMD-QoL has demonstrated superior content validity compared to both EQ-5D and PROMIS. Its ability to capture the multifaceted effects of DMD on patients’ physical, psychological, and social well-being highlights the importance of developing a similar SMA-specific PBM in the future. Such a measure can provide a comprehensive and accurate assessment of the impact of SMA on patients’ QoL. This will enable healthcare professionals and researchers to better understand the unique challenges faced by individuals with SMA, leading to improved patient care and the development of targeted interventions.

Additionally, while the known-group test has demonstrated that EQ-5D and PROPr can differentiate between SMA risk groups, the responsiveness, as one of most important characteristics of patient-reported outcome measures (PROMs) in the clinical practice, was not assessed. PROMs are used to capture the patient’s perspective over time and offer valuable insights into their experiences and well-being. PROMs with good responsiveness can help evaluate the effectiveness of healthcare interventions, inform clinical decision-making, and contribute to patient-centered care [42, 48]. The responsiveness of the two instruments should be explored in the future.

The primary limitation of this study is that the PROPr was developed based on the US value set, which might result in a systematic bias in assessing its psychometric properties because the PROPr does not yet incorporate a Chinese value set. We have further conducted analyses using the US EQ-5D-5L utility score and the performance improved compared to the China utility score (Appendix, Tables A2-A4). It indicates that cultural heterogeneity may exist. Individuals from different cultural backgrounds may perceive and value HRQoL differently when assessed using the EQ-5D instrument. Thus, Chinese PROPr utility score is expected in the future. The second limitation of this study is that all data used were self-reported, which could introduce recall bias. Self-reported bias can be either random or systematic, and may lead to various issues in interpreting the findings. In particular, previous studies have highlighted concerns regarding the content validity of the EQ-5D in certain patient groups, potentially resulting in patients having a different understanding of how to measure their HRQoL. Third, another limitation of the study is the imbalance in the sample distribution (SMA 2/3 > 75%, Appendix Tables A5-A8 show the measurement properties of patients with SMA 1), which may impactthe generalizability of the findings and limit the ability to draw conclusions that apply to a broader SMA patient group. It would have been beneficial to include a more diverse sample of participants to ensure a more representative representation in the future. Lastly, this study does not assess other important psychometric properties, such as face validity and criterion validity, of the EQ-5D and PROMIS. This limitation may affect the generalizability of our findings.

## Conclusions

Our results showed the high validity of both EQ-5D-5L and PROPr assessed using PROMIS-29 to support an economic evaluation of SMA-related health techniques or rehabilitation interventions. EQ-5D-5L generated a slightly higher utility score than PROPr but exhibited high floor effects and sufficient ceiling effects, whereas most dimensions of PROMIS-29 had neither ceiling nor floor effects. Additionally, PROPr showed a stronger correlation with SMA daily activities but a poorer discriminant ability to differentiate individuals from symptom groups than the EQ-5D-5L. Therefore, the selection of PROPr and EQ-5D-5L should depend on the nature of the intervention in SMA.

### Electronic supplementary material

Below is the link to the electronic supplementary material.

Supplementary Material 1

## Data Availability

Derived data supporting the findings of this study are available from the corresponding author on request.
