# Supplementary material for: Measurement properties of the EQ-5D-5L and PROPr in patients with spinal muscular atrophy
Source: Health Qual Life Outcomes. 2023 Nov 15;21:123. doi: 10.1186/s12955-023-02204-z (PMC10647137; doi:10.1186/s12955-023-02204-z)
Supplement: Supplementary file 1 — Supplementary Material 1 [file 12955_2023_2204_MOESM1_ESM.docx]

Table A1 Known-group validity of the EQ-5D and PROPr utility score

|  | **N (%)** | **EQ-5D utility score** | | **PROPr** | |
| --- | --- | --- | --- | --- | --- |
|  |  | **Mean (SD)** | **p-value** | **Mean (SD)** | **p-value** |
| Shortness of breadth |  |  |  |  |  |
| No/minor | 51(37.2) | 0.36(0.2) | <0.001 | 0.29(0.09) | <0.001 |
| Moderate/Severe | 86(62.8) | 0.22(0.25) |  | 0.18(0.18) |  |
| Effect Size^&^ |  | 0.29 |  | 0.33 |  |
| Ulcer/Rash |  |  |  |  |  |
| No/minor | 42(30.7) | 0.4(0.2) | <0.001 | 0.28(0.1) | 0.001 |
| Moderate/Severe | 95(69.3) | 0.21(0.24) |  | 0.18(0.17) |  |
| Effect Size |  | 0.37 |  | 0.28 |  |
| Leg pain |  |  |  |  |  |
| No/minor | 67(48.9) | 0.35(0.22) | <0.001 | 0.29(0.12) | <0.001 |
| Moderate/Severe | 70(51.1) | 0.19(0.24) |  | 0.2(0.18) |  |
| Effect Size |  | 0.33 |  | 0.28 |  |
| Stiff back |  |  |  |  |  |
| No/minor | 98(71.5) | 0.33(0.23) | <0.001 | 0.32(0.12) | 0.005 |
| Moderate/Severe | 39(28.5) | 0.13(0.22) |  | 0.22(0.21) |  |
| Effect Size |  | 0.37 |  | 0.24 |  |
| Hand weakness |  |  |  |  |  |
| No/minor | 101(73.7) | 0.3(0.22) | 0.018 | 0.38(0.12) | <0.001 |
| Moderate/Severe | 36(26.3) | 0.18(0.28) |  | 0.2(0.19) |  |
| Effect Size |  | 0.2 |  | 0.2 |  |
| Dysphagia |  |  |  |  |  |
| No/minor | 36(26.3) | 0.4(0.19) | <0.001 | 0.27(0.11) | 0.007 |
| Moderate/Severe | 101(73.7) | 0.23(0.25) |  | 0.19(0.17) |  |
| Effect Size |  | 0.31 |  | 0.23 |  |
| SMA Type |  |  |  |  |  |
| Type I | 58(42.3) | 0.34(0.2) | 0.01 | 0.36(0.13) | 0.01 |
| Type II | 76(55.5) | 0.22(0.27) |  | 0.27(0.17) |  |
| Type III | 3(2.2) | 0.1(0.16) |  | 0.21(0.2) |  |
| Effect Size |  | 0.05 |  | 0.04 |  |
| Nusinersen |  |  |  |  |  |
| Yes | 79 (57.7) | 0.25(0.26) | 0.31 | 0.22(0.16) | 0.008 |
| No | 58(42.3) | 0.30(0.23) |  | 0.27(0.16) |  |
| Effect Size |  | 0.001 |  | 0.04 |  |

& < 0.3 small effect; 0.30 < 0.5 moderate effect; and ≥ 0.5 large effect.

Table A2 convergent validity between EQ-5D-5L and PROMIS using HK and US value

| **Items/Dimensions** | **Correlation coefficient (95% C.I.)** | |
| --- | --- | --- |
|  | **China utility score** | **US utility score** |
| **EQ-5D Utility score, PROPr and SMAIS** |  |  |
| EQ-5D Utility score vs. SMAIS | 0.28 (0.11, 0.41) ^***^ | 0.35 (0.2, 0.5) ^***^ |
| **Agreement between EQ-5D utility score and PROPr (ICC)** |  |  |
| EQ-5D Utility score vs. PROPr | 0.4 (0.31,0.57) | 0.49 (0.35,0.61) |

* p<0.05; ** p<0.01; *** p<0.001

Table A3 linear regression analysis of EQ-5D utility score by HK and US value

|  | EQ-5D utility score model | |
| --- | --- | --- |
|  | China value | US value |
| EQ-5D Utility score | -13.05(-29.66,3.56) | -14.02 (-27.45,-0.59) |
| Gender - Male | -2.35(-10.54,5.83) | -2.7 (-10.84, 5.44) |
| Age | -0.11(-0.71,0.49) | -0.13 (-0.72,0.47) |
| Duration | -0.05(-0.46,0.35) | -0.05 (-0.45, 0.35) |
| Type T2 | -6.72(-15.75,2.31) | -7.64 (-16.71, 1.42) |
| Type T3 | 20.66(-7.6,48.93) | 19.89 (-8.15, 47.93) |
|  |  |  |
| Adjusted R^2^ | 0.03 | 0.04 |
| BIC | 1273.56 | 1271.66 |

Table A4 known-group validity between HK and US EQ-5D utility score

|  | **China value** | | **US value** | |
| --- | --- | --- | --- | --- |
|  | **Mean (SD)** | **p-value** | **Mean (SD)** | **p-value** |
| Shortness of breadth |  |  |  |  |
| No/minor | 0.36(0.2) | <0.001 | 0.33(0.26) | <0.001 |
| Moderate/Severe | 0.22(0.25) |  | 0.12(0.31) |  |
| Effect Size | 0.29 |  | 0.32 |  |
| Ulcer/Rash |  |  |  |  |
| No/minor | 0.4(0.2) | <0.001 | 0.37(0.25) | <0.001 |
| Moderate/Severe | 0.21(0.24) |  | 0.12(0.3) |  |
| Effect Size | 0.37 |  | 0.4 |  |
| Leg pain |  |  |  |  |
| No/minor | 0.35(0.22) | <0.001 | 0.3(0.27) | <0.001 |
| Moderate/Severe | 0.19(0.24) |  | 0.1(0.31) |  |
| Effect Size | 0.33 |  | 0.34 |  |
| Stiff back |  |  |  |  |
| No/minor | 0.33(0.23) | <0.001 | 0.27(0.29) | <0.001 |
| Moderate/Severe | 0.13(0.22) |  | 0.02(0.28) |  |
| Effect Size | 0.37 |  | 0.38 |  |
| Hand weakness |  |  |  |  |
| No/minor | 0.3(0.22) | 0.018 | 0.25(0.29) | 0.002 |
| Moderate/Severe | 0.18(0.28) |  | 0.06(0.33) |  |
| Effect Size | 0.2 |  | 0.26 |  |
| Dysphagia |  |  |  |  |
| No/minor | 0.4(0.19) | <0.001 | 0.37(0.25) | <0.001 |
| Moderate/Severe | 0.23(0.25) |  | 0.14(0.3) |  |
| Effect Size | 0.31 |  | 0.34 |  |
| SMA Type |  |  |  |  |
| Type I | 0.34(0.2) | 0.01 | 0.31(0.26) | 0.001 |
| Type II | 0.22(0.27) |  | 0.12(0.32) |  |
| Type III | 0.1(0.16) |  | 0.01(0.21) |  |
| Effect Size | 0.05 |  | 0.09 |  |
| Nusinersen |  |  |  |  |
| Yes | 0.25(0.26) | 0.31 | 0.16(0.32) | 0.1 |
| No | 0.30(0.23) |  | 0.25(0.28) |  |
| Effect Size | 0.001 |  | 0.01 |  |

Table A5 Profile of EQ-5D and PROMIS-29 for SMA type 1

| **Variable** | **Ceiling effect (%)** | **Floor effect (%)** | **Mean** | **Standard deviation** | **Median** | **Range** |
| --- | --- | --- | --- | --- | --- | --- |
| **EQ-5D-5L dimensions** |  |  |  |  |  |  |
| Mobility | 0 | 94.8 | - | - | - | - |
| Self-care | 86.2 | 1.7 | - | - | - | - |
| Usual activities | 53.4 | 0 | - | - | - | - |
| Pain/discomfort | 32.8 | 32.8 | - | - | - | - |
| Anxiety/Depression | 34.5 | 24.1 | - | - | - | - |
| Best health (11111) | 0.0 | - | - | - | - | - |
| Worst health (55555) | - | 0.0 | - | - | - | - |
| **EQ-5D utility score** | 1.7 | 6.9 | 0.34 | 0.2 | 0.32 | -0.22 – 0.66 |
| **EQ-VAS** | 1.7 | 3.4 | 60.5 | 22.8 | 69.5 | 7~100 |
|  |  |  |  |  |  |  |
| **PROMIS-29 subscales** |  |  |  |  |  |  |
| Anxiety/Fear | 1.7 | 17.4 | 57.9 | 9.6 | 57.6 | 40.3 – 75.5 |
| Cognition | 1.7 | 1.7 | 48.2 | 9.6 | 46 | 29.6 – 66.8 |
| Depression/Sadness | 1.7 | 22.4 | 57.5 | 10.9 | 58.9 | 41 – 79.3 |
| Fatigue | 3.4 | 5.2 | 54.7 | 9.6 | 55.2 | 33.7 – 75.8 |
| Pain intensity VAS | 17.2 | 1.7 | 3.9 | 2.4 | 3.5 | 1 – 10 |
| Pain interference | 1.7 | 32.7 | 53.6 | 10.2 | 55.6 | 41.6 – 75.6 |
| Physical function | 1.7 | 68.9 | 24.9 | 5.2 | 22.6 | 22.6 – 57 |
| Sleep disturbance | 1.7 | 5.2 | 49.3 | 7.2 | 50.6 | 32 – 60.5 |
| Social roles and activities | 5.2 | 22.4 | 39.8 | 10.6 | 37.2 | 27.5 – 64.2 |
| **PROPr** | 1.7 | 3.4 | 0.21 | 0.13 | 0.17 | 0.05 – 0.6 |

Table A6 convergent validity between EQ-5D-5L and PROMIS for SMA type 1

| **Items/Dimensions** | **Correlation coefficient**  **(95% C.I.)** |
| --- | --- |
| **EQ-5D dimensions and PedsQL core questionnaire** | |
| EQ-5D Mobility vs. PedsQL Physical functioning | -0.47(-0.59,-0.33) ^***^ |
| EQ-5D Usual Activity vs. PedsQL Social functioning | -0.31(-0.45,-0.15) ^***^ |
| EQ-5D Pain/discomfort vs. PedsQL item hurt or ache | -0.22 (-0.19,-0.03) ^***^ |
| EQ-5D Anxiety/depression vs. PedsQL Emotional functioning | -0.25(-0.38,-0.08) ^*^ |
| EQ-5D Self-care vs. PedsQL item hard to take bath or shower | -0.78(-0.89,-0.53) ^***^ |
| **PROMIS-29 and PedsQL core questionnaire** | |
| PROMIS Physical function vs. PedsQL Physical functioning | 0.69(0.59,0.76) ^***^ |
| PROMIS Fatigue vs. PedsQL physical functioning | -0.40(-0.53,-0.25）^***^ |
| PROMIS Anxiety/Fear vs. PedsQL Emotional functioning | -0.81 (-0.88, -0.70) ^***^ |
| PROMIS Depression/Sadness vs. PedsQL Emotional functioning | -0.78 (-0.87, -0.66) ^***^ |
| PROMIS Cognition vs. PedsQL Emotional functioning | 0.82(0.72,0.89) ^***^ |
| PROMIS Social roles and activities vs. PedsQL Social functioning | 0.61(0.41,0.75) ^***^ |
| PROMIS Pain intensity VAS vs. PedsQL item hurt or ache | -0.42 (-0.54, -0.20) ^***^ |
| PROMIS Pain interference vs. PedsQL item hurt or ache | -0.46 (-0.61, -0.33) ^***^ |
| PROMIS Sleep disturbance vs. PedsQL item Trouble sleeping | -0.69 (-0.72, -0.61) ^***^ |
| **EQ-5D Utility score, PROPr and SMAIS** |  |
| EQ-5D Utility score vs. SMAIS | 0.22(0.04,0.45) ^*^ |
| PROPr vs. SMAIS | 0.17(0.08,0.42) ^*^ |
| **EQ-5D dimensions and PROMIS-29** |  |
| EQ-5D Mobility vs. PROMIS Physical function | -0.23(-0.46, -0.03) ^*^ |
| EQ-5D Self-care vs. PROMIS Physical function | 0.06(-0.11,0.35) |
| EQ-5D Usual activities vs. PROMIS Social roles and activities | -0.2(-0.05,0.44) |
| EQ-5D Pain/discomfort vs. PROMIS Pain intensity VAS | 0.33(0.11,0.50) ^***^ |
| EQ-5D Pain/discomfort vs. PROMIS Pain interference | 0.42(0.18,0.60) ^***^ |
| EQ-5D Anxiety/depression vs. PROMIS Anxiety/Fear | 0.06(-0.21,0.33) |
| EQ-5D Anxiety/depression vs. PROMIS Depression/Sadness | 0.05(-0.22,0.30) |
| **Agreement between EQ-5D utility score and PROPr (ICC)** |  |
| EQ-5D Utility score vs. PROPr | 0.3(0.05,0.52)^*^ |

* p<0.05; ** p<0.01; *** p<0.001

Table A7 Linear regression analysis between EQ-VAS and EQ-5D and PROMIS adjusted by demographics for SMA type 1

|  | EQ-5D utility score model | EQ-5D dimension model | PROPr  model | PROMIS dimension model |
| --- | --- | --- | --- | --- |
| EQ-5D |  |  |  |  |
| Utility score | -1.73(-37.8,34.7) | - | - | - |
| Mobility | - | -2.24(-17.45,12.96) | - | - |
| Self-care | - | 1.49(-7.88,10.86) | - | - |
| Usual activities | - | 4.47(-2.69,11.63) | - | - |
| Pain/Discomfort | - | -1.65(-6.28,2.98) | - | - |
| Anxiety/depression | - | 1.56(-3.53,6.65) | - | - |
| PROMIS |  |  |  |  |
| PROPr | - | - | 40.26(-12.33,92.85) | - |
| Anxiety/Fear | - | - | - | 22.88(-106.85,152.62) |
| Cognition | - | - | - | 4.81(-16.71,26.33) |
| Depression/Sadness | - | - | - | -12.27(-80.99,56.45) |
| Fatigue | - | - | - | -1.05(-2.25,0.15) |
| Pain intensity VAS | - | - | - | 18.05(-79.7,115.8) |
| Pain interference | - | - | - | -5.14(-34.82,24.53) |
| Physical function | - | - | - | -0.7(-1.85,0.45) |
| Sleep disturbance | - | - | - | 39.99(-176.27,256.24) |
| Social roles and activities | - | - | - | 10.51(-24.86,45.88) |
| Gender - Male | -3.46(-11.56,4.64) | -5.14(-21.1,10.82) | -8.21(-23.05,6.62) | -9.51(-25.11,6.08) |
| Age | -0.12(-0.71,0.47) | -0.05(-1.3,1.21) | -0.06(-1.24,1.11) | 0.06(-1.12,1.25) |
| Duration | -0.18(-0.57,0.22) | -0.59(-1.46,0.27) | -0.33(-1.15,0.48) | -0.11(-0.95,0.74) |
|  |  |  |  |  |
| Adjusted R^2^ | -0.02 | -0.04 | 0.02 | 0.1 |
| BIC | 553.133 | 566.07 | 550.61 | 568.75 |

Table A8 Known-group validity of the EQ-5D and PROPr utility score

|  | **N (%)** | **EQ-5D utility score** | | **PROPr** | |
| --- | --- | --- | --- | --- | --- |
|  |  | **Mean (SD)** | **p-value** | **Mean (SD)** | **p-value** |
| Shortness of breadth |  |  |  |  |  |
| No/minor | 32(55.2) | 0.39(0.17) | 0.05 | 0.28(0.16) | 0.002 |
| Moderate/Severe | 26(44.8) | 0.28(0.22) |  | 0.16(0.08) |  |
| Effect Size^&^ |  | 0.25 |  | 0.41 |  |
| Ulcer/Rash |  |  |  |  |  |
| No/minor | 18(31) | 0.41(0.15) | 0.06 | 0.24(0.14) | 0.02 |
| Moderate/Severe | 40(69) | 0.31(0.21) |  | 0.15(0.05) |  |
| Effect Size |  | 0.24 |  | 0.31 |  |
| Leg pain |  |  |  |  |  |
| No/minor | 29(50) | 0.40(0.19) | 0.03 | 0.25(0.14) | 0.01 |
| Moderate/Severe | 29(50) | 0.28(0.21) |  | 0.17(0.11) |  |
| Effect Size |  | 0.27 |  | 0.32 |  |
| Stiff back |  |  |  |  |  |
| No/minor | 44(75.9) | 0.38(0.12) | 0.006 | 0.26(0.17) | 0.18 |
| Moderate/Severe | 14(24.1) | 0.2(0.09) |  | 0.20(0.12) |  |
| Effect Size |  | 0.36 |  | 0.18 |  |
| Hand weakness |  |  |  |  |  |
| No/minor | 52(89.6) | 0.35(0.18) | 0.3 | 0.19(0.12) | 0.008 |
| Moderate/Severe | 6(10.4) | 0.23(0.28) |  | 0.37(0.13) |  |
| Effect Size |  | 0.14 |  | 0.34 |  |
| Dysphagia |  |  |  |  |  |
| No/minor | 29(50) | 0.38(0.18) | 0.14 | 0.17(0.11) | 0.03 |
| Moderate/Severe | 29(50) | 0.3(0.21) |  | 0.25(0.15) |  |
| Effect Size |  | 0.19 |  | 0.28 |  |
| Nusinersen |  |  |  |  |  |
| Yes | 31 (53.4) | 0.36(0.19) | 0.49 | 0.24(0.13) | 0.04 |
| No | 27(46.6) | 0.32(0.2) |  | 0.19(0.14) |  |
| Effect Size |  | 0.01 |  | 0.05 |  |

& < 0.3 small effect; 0.30 < 0.5 moderate effect; and ≥ 0.5 large effect.
